# Neobladder “Function”: Tips and Tricks for Surgery and Postoperative Management

**DOI:** 10.3390/life12081193

**Published:** 2022-08-04

**Authors:** Daniela Fasanella, Michele Marchioni, Luigi Domanico, Claudia Franzini, Antonino Inferrera, Luigi Schips, Francesco Greco

**Affiliations:** 1 Urology Unit, Department of Medical, Oral and Biotechnological Sciences, G. d’Annunzio University of Chieti, SS Annunziata Hospital, 66100 Chieti, Italy; 2 Urology Unit, Centro Salute Uomo, Via Palma il Vecchio 4a, 24122 Bergamo, Italy

**Keywords:** bladder cancer, orthotopic neobladder, robot-assisted radical cystectomy, urinary diversion, complications, continence, oncological outcomes, functional outcomes, quality of life

## Abstract

Orthotopic neobladder (ONB) reconstruction is a continent urinary diversion procedure increasingly used in patients with muscle-invasive bladder cancer following radical cystectomy (RC). It represents a valid alternative to the ileal duct in suitable patients who do not prefer a stoma and are motivated to undergo adequate training of the neobladder. Careful patient selection, taking into account the absolute and relative contraindications for ONB as well as an adequate recovery protocol after surgery are integral to the success of this procedure and the oncological and functional outcomes. The objective of this review is to summarize the current data on RC with ONB in terms of patient selection, preoperative preparation, surgical techniques and functional (continence and sexual activity) and oncological outcomes, with particular attention to the management of complications and the impact on quality of life (QoL).

## 1. Introduction

Radical cystectomy (RC) with lymph-node dissection and urinary diversion (UD) is the standard treatment for muscle-invasive and for selected high-risk nonmuscle invasive bladder cancer failing bladder-sparing therapy [1]. The surgical approach has evolved over time, up to the adoption of robot-assisted techniques. The type of urinary diversion performed depends on the patient’s and surgeon’s preferences, associated comorbidities and postoperative quality of life (QoL).

Orthotopic neobladder (ONB) allows voiding through the native urethra, removing the need for stoma appliances or catheters without compromising cancer control [2]. While there are various urinary diversion procedures, the neobladder may be the preferred option for many reasons. First of all, it allows the patient to be continent, ensuring him to control his urinary function by maintaining body image and obtaining a better QoL than other noncontinent urinary diversions, such as ileal conduit [3] (IC). However, not all patients may be suitable for this type of reconstruction. A careful global evaluation of the patient is required, including his basic characteristics, comorbidities, continence and the features of his intestinal and urinary systems.

The aim of this review is to explore the various aspects of ONB function, from the surgical approaches to the functional outcomes and postoperative management.

## 2. Orthotopic Neobladder

### 2.1. Literature Search Strategy

A literature review was conducted by consulting several databases (PubMed, MEDLINE, Research Gate and Google Scholar) and using the following keywords: “orthotopic neobladder,” “radical cystectomy,” “continence,” “complications,” “oncologic outcomes,” “functional outcomes” and “quality of life.” The articles selected and cited in this review were searched essentially within the last 20 years, focusing mainly on the most recent papers. The choice to include some older articles was related to their relevance to the treatment of the topic. All study designs and publication types (reviews, narratives, or trials) were considered. Articles related to search terms were evaluated through titles, abstracts and content. The review was completed by manually analyzing the bibliographic references of the various selected articles.

### 2.2. Patient Selection and Preoperative Management

Radical cystectomy and urinary diversion are the two phases of a single operation. As underlined by the European Guidelines, the literature reports the complications of RC, ignoring the fact that most of them are related to diversion [1,4]. Age alone is not the only criterion to be taken into consideration in selecting patients to whom continent diversion is proposed (in patients with age > 80 years it is not recommended) [5,6]. It is important to evaluate comorbidities, cardiac, pulmonary and cognitive functions and tumor features [7] as well as patient preferences. In fact, the patient must be strongly motivated to know their urinary diversion and how to manage it in the most appropriate way [8,9].

Certainly, the main contraindications to neobladder reconstruction include: limited life expectancy, debilitating neurological and psychiatric pathologies, a severe inflammatory bowel disease, impaired liver or kidney [10] and positive surgical margins for urothelial carcinoma [11]. Other relative contraindications are high-dose preoperative radiotherapy, the presence of complex urethral stricture and severe urethral sphincter incontinence [5] (Table 1).

To improve the patient selection process, studied frailty indices have also been proposed that can be used to identify patients most suitable for orthotopic neobladder reconstruction [12], as they would be able to better predict the risk of postoperative complications than age, body mass index (BMI), or ASA score. At present, these indices should be further investigated for their role in patient selection [13].

Fragility, advanced age and comorbidities are nonoverlapping patient characteristics. Of these, frailty represents the most consistent and strongest predictor of early adverse outcomes after CR [14]. Frailty represents a state of health characterized by reduced physical reserve and greater vulnerability to stress factors, and it is the main predictor of complications, failure to rescue (FTR), in-hospital mortality, length of stay (LOS) and total hospital charges (THCs) [14,15]. Consequently, presurgical recognition of frail patients should be included in clinical practice in order to refer these patients to prehabilitation programs (nutrition, exercise and psychological support) that can improve perioperative outcomes [16]. It must also be considered that often the underlying disease may not allow adequate time for such measures to be implemented before the cystectomy. For this reason, it is important to have an exhaustive discussion about the goals of care and the most suitable surgical choices for the patient.

Standard preoperative management includes computed tomography (CT) of the chest, abdomen and pelvis, blood tests and anesthetic evaluation. In robotic-assisted radical cystectomy (RARC) with intracorporeal urinary reconstruction, bowel preparation is not necessary [17,18,19]. Preoperative antibiotics and venous thromboembolism (VTE) prophylaxis are administered [20,21].

Radical cystectomy with the creation of a neobladder is a complex procedure characterized by potentially high morbidity [22]. For this reason, the Enhanced Recovery After Surgery (ERAS) protocols have been introduced in order to improve perioperative outcomes and reduce complications and hospital stay in patients undergoing RC [23,24,25]. Initially applied to colorectal surgery, ERAS protocols have gradually been adopted by other types of surgery as well, including urology [26,27]. Recommended by the EAU [1] and AUA [28] guidelines for RC with UD, ERAS protocols still do not represent the standard of care [29,30]. There are still few findings in the literature regarding patients who have undergone RC, but the available studies have yielded positive results [19,31,32,33,34,35].

The ERAS protocol represents a set of multimodal interventions concerning the preoperative, intraoperative and postoperative period. It consists of 34 themes [18], including anaesthesia, analgesia, nutritional status, perioperative fluid management, early mobilization, prevention of hypothermia and deep vein thrombosis, antimicrobial prophylaxis, prevention of postoperative ileus (POI) and early oral diet [27,36]. Recent studies suggest that the length of hospital stay (LOS) and the risk of POI are statistically significantly lower in patients undergoing the ERAS protocol than in those using the traditional approach, without an increase in morbidity or hospital readmission rate [37,38].

For the management of postoperative pain, a reduction in the use of opioids is recommended, with the use of high-dose paracetamol and/or ketorolac. Xu et al. [39] showed that patients subjected to the ERAS protocol felt more pain than patients subjected to a traditional protocol but had a lower rate of postoperative ileus.

A relevant issue regarding ERAS is the high variability between published protocols, such as to make it difficult for doctors to identify the most appropriate measures for correct patient management. These protocols differ in the number and modality of the ERAS elements. Although there are protocol-specific recommendations for patients undergoing RC [27], most of them are evidence-based and sometimes the level of this evidence is low. This remarkable variability in protocols can be found in the prevention of paralytic ileus. The recovery of intestinal function can be favored by early mobilization, early oral feeding, use of metoclopramide or alvimopan [40] and chewing gums [41].

Wessels et al. [42] highlighted that 52% of the protocols analyzed included more than one of these elements to prevent postoperative ileus, but each protocol nevertheless presented a different approach. This variability can also be highlighted in the optimized management of fluids in RC, which can lead to a benefit for gastrointestinal function [43]. However, also for this element in the various protocols different strategies have been described, such as for monitoring the volume status.

Certainly, elements to be taken into consideration are: an adequate carbohydrate load, which would lead to a reduction in LOS and insulin resistance [44]; early mobilization, with a reduction in pulmonary and thromboembolic complications [45]; and adequate antibiotic prophylaxis, even if at the moment there are no clear recommendations regarding the duration and type of antibiotic to be inserted preventively [46].

Although there are differences in the pathways, in particular regarding which elements to adopt universally, the use of the ERAS protocols has shown a significant improvement in the perioperative outcomes of patients undergoing RC compared with patients with non-ERAS perioperative care [47,48]. However, in order to cope with this considerable variability, further clinical evidence and randomized studies are necessary to confirm the results present to date and for a better definition of the individual ERAS protocols.

### 2.3. Which Neobladder?

Several types of orthotopic neobladders were made years before the advent of the robotic era. The goal was to provide a reservoir that was as similar as possible to the original bladder. The intestinal segment most used is the terminal ileum, as it is more extensible and has a greater capacity than the other intestinal segments, favoring the conservation of urine at a lower pressure, avoiding the risk of kidney damage, metabolic consequences and incontinence [49]. It is also important to carry out the detubulation of the terminal ileum, constructing a possibly spherical reservoir with a greater radius in order to contain a higher volume at a lower pressure, according to Laplace’s law [50].

Different types of neobladders have been created: Camey I and II (U-shaped), modified Camey II (Z-shaped), Kock-pouch, T-Pouch, Studer clutch bag, Hautmann (W-shaped), Abol-Enein and Ghoneim modification of the W pouch and the Vescica Ileale Padovana (VIP, circularly shaped). The introduction of RARC by Menon in 2003 led to a new era in pelvic cancer surgery [51]. Robotic-assisted radical cystectomy with extracorporeal urinary diversion (ECUD) is a hybrid technique that has been increasingly employed as an approach intended to improve perioperative recovery, minimizing pain and complications compared with open radical cystectomy (ORC) [52]. Furthermore, the oncological results appear to be comparable for both techniques [53]. In recent years, with the rise of RARC, intracorporeal urinary diversion (ICUD) is becoming increasingly popular as a viable alternative to ECUD, both for cancer and survival outcomes, as well as for functional and urodynamic results [54]. In the literature, several authors have reported their experience regarding RARC-ICUD, exposing preliminary oncological and functional results that are entirely satisfactory and promising, although further studies are needed for a long-term evaluation of functional outcomes [7,17,54,55,56,57].

### 2.4. Neobladder Complications

More than half of patients undergoing orthotopic reconstruction experience complications within 90 days of surgery [4]. Most are secondary to sequelae of the urinary and intestinal tracts. In the immediate postoperative period, the most frequent complications are urinary tract infections or intra-abdominal abscesses. General, genitourinary (hydronephrosis), gastrointestinal (ileus, intestinal obstruction) complications, wound infection and/or dehiscence, ureteroileal stenosis, neobladder fistulas and spontaneous neobladder rupture may occur [58,59].

The most common gastrointestinal complication is paralytic ileus: patients are best treated with hydration, use of nasogastric tube, resolution of electrolyte imbalances and discontinuation of medications [60]. Perioperative use of alvimopan, a peripheral μ-opioid receptor antagonist, would appear to promote recovery of bowel function, decreasing POI rate and reducing LOS [40,61,62].

Patients undergoing the creation of a neobladder are susceptible to developing intra-abdominal, urinary and wound infections due to the use of the bowel. Treatment includes early initiation of intravenous fluid administration, broad-spectrum antibiotics and skin sterilization with chlorhexidine [63,64]. The choice of the most appropriate antibiotic should also be based on the results of culture tests and antibiogram [65].

The most common genitourinary complications in the immediate postoperative period are urinary leakage and ureteral obstruction [66,67]. A urine leakage can occur at the uretero-intestinal anastomosis or urethra-neobladder anastomosis, and it may manifest as increased intraperitoneal drainage, elevated serum creatinine, or even chemical peritonitis and ileus. Normally, the use of percutaneous drainage, nephrostomy or intraoperative ureteral stents allows effective treatment of leakage [68,69]. Ureteral obstruction may occur due to edema or scarring at the uretero-intestinal anastomosis and may present clinically with flank pain, acute pyelonephritis, or altered renal function indices in the absence of symptoms. Resolution of the obstructive process is necessary in these cases, with immediate placement of a nephrostomy tube and subsequent careful evaluation of the uretero-intestinal anastomosis for possible stenosis.

Sometimes, in up to 14% of cases, a second surgical procedure may be necessary for intestinal obstruction, wound dehiscence, abscess or lymphocele drainage, continent pocket rupture and intestinal or vaginal fistulas [67,70,71]. Contrast imaging studies are critical to confirm the diagnosis. Furthermore, from 3% to 9% of patients have a mortality within 90 days of surgery and the most frequent causes are represented by sepsis and cardiovascular complications [4,67,70,72].

Hosseini et al. [73] studied the stenosis rates of benign ureterointestinal anastomosis in 369 patients undergoing RARC with ICUD between 2003 and 2015. The overall stenosis rate was 6.5% for a median follow-up time of 33 months. Parekh et al. [53] presented the results of the RAZOR (Randomized Open versus Robotic Cystectomy) trial, a randomized, open-label, phase 3 noninferiority study conducted in 15 medical centers in the United States, comparing RARC with ECUD and ORC, reporting no significant differences in length of stay and complications between the two techniques.

To date, several approaches are available for the management of neobladder complications, but endoscopic management is recommended first, if possible [74]. With stenosis of the uretero-intestinal anastomosis and between the urethra and the neobladder, stones and tumor recurrence of the neobladder can be managed endoscopically [75].

With regard to uretero-intestinal stenosis, balloon dilation or endoscopic incision can be attempted by cold knife or laser incision. Success rates reach 70%, but the determining factors for the success of endoscopic treatment are the length and the side on which the stenosis occurs. In fact, the treatment of a stenosis longer than 1 cm, which occurs within the first 6 months after surgery and localized to the left side, is characterized by poor results [74,76].

Neobladder stones are a rare and late complication. Predisposing factors include mucus overproduction, metabolic acidosis, the presence of metal staples, urinary infections and ineffective emptying [77,78]. Neobladder stones can be treated with shock wave lithotripsy, endoscopically or percutaneously, or with laparoscopic and open surgery [79,80,81]. Long-term complications can also verify, with an overall rate of 40.8%, as the outcomes of previous treatments for bladder cancer, such as radiotherapy [82].

### 2.5. Electrolyte Disorders and Nutrition

Due to the greater permeability of the intestinal mucosa, the neobladder leads to alterations in the absorption of electrolytes (potassium, hydrogen and chlorine ions), leading to hyperkalaemic hyperchloremic metabolic acidosis, which must be carefully monitored for the entire postoperative period, as up to about half of patients may detect metabolic acidosis 1 month after the procedure [83]. Chronic metabolic acidosis can over time lead to calcium reabsorption and decreased circulating phosphate levels, leading to osteomalacia and osteoporosis [84]. An elevated urinary calcium level secondary to metabolic acidosis may also predispose patients with ONB to an increased risk of urinary stones [85], often composed of struvite, secondary to organisms such as the Proteus and Klebsiella species [86]. This also carries an increased risk of urinary tract infections [87]. Bicarbonate supplementation is essential for correcting acidosis and may be needed in the long term in up to 33% of patients [59].

Patients undergoing neobladder formation have a higher risk of developing paralytic ileus in the first 5 days after surgery than those undergoing ileal conduit [88]. A careful monitoring and integration of vitamin B12 and folic acid absorbed at the level of the terminal ileum is essential. Terminal ileal reduction also interferes with the reabsorption of bile acids, resulting in fat malabsorption that can precipitate diarrhea and dehydration [89].

### 2.6. Oncological Outcomes

Early recurrence is an unfavorable prognostic factor, correlating closely with recurrence-free survival (RFS) and 5-year overall survival (OS) [90]. To date, the most recent evidence in the literature regarding long-term outcomes after RARC shows oncologic outcomes fully comparable to those in open series [91]. RFS, CSS and OS have been documented as similar in all RCTs, including the largest RAZOR trial (*n* = 302) [92]. The RAZOR trial evidenced that RARC is not inferior in terms of 2-year PFS (progression-free survival) (72.3% vs. 71.6%), AEs (67% vs. 69%) and QoL as compared with open cystectomy [53]. Even in a systematic review by the Pasadena Consensus Panel (PCP), RARC appears to be broadly equivalent to ORC in terms of morbidity and mortality, oncologic outcomes and complication rates [93].

As indicated by Raza et al., other factors associated with early recurrence include lymph node involvement, advanced pathologic stage and positive surgical margin rates [91]. A multicenter RARC series published by the EAU Scientific Working Group (ESWG) showed that lymph-node-positive, non-organ-confined disease and positive surgical margin (PSM) were associated with oncologic outcomes, indicating that tumor recurrence following RARC is primarily related to tumor biology and not to surgical treatment modality [94]. In this way, early and late cancer outcomes are encouraging, and this represents a further reason for the adoption of RARC.

A positive surgical margin rate after RC has been described in the literature ranging from 4 to 15% [95,96]. The finding of ureteral, urethral and soft tissue PSM carries a sevenfold increase in the risk of developing urothelial recurrence [97]. Claps et al. [98], in their study, found that the 3-year disease-specific survival (DSS) of ureteral PSMs was no different from negative surgical margins, but urethral and soft tissue PSMs showed worse rates of DSS. European guidelines recommend carefully evaluating possible involvement of the prostatic urethra at the time of primary TURB or from the frozen section during the cystoprostatectomy procedure, before performing an orthotopic reconstruction [1]. A negative frozen section of the urethra can reliably identify patients in whom urethrectomy should be avoided, while the finding of a urethral tumor requires a urethrectomy, as well as being a contraindication to ONB [1,99].

In the multicenter study conducted by Laukhtina et al. [100], urethrectomy does not appear to improve survival outcomes (PFS, CSS, OS) in all patients treated with RC for urothelial bladder cancer. However, there was evidence of improvement in PFS after urethrectomy at the time of RC in patients at high risk of urothelial recurrence (papillary pattern, multiple tumors and/or concomitant CIS), as reported in a previous study by Hakozaki et al. [101]. This is why adequate stratification based on risk factors is important and necessary in order to select patients who bring benefit from immediate urethrectomy during RC.

Another element to consider is the finding of incidental prostate cancer (iPCa) at the time of RC. In previous series, a prevalence rate from 4% up to 61% has been reported, with a notable difference in the results and clinical characteristics [102,103]. As reported by Fahmy et al. [104], iPca was significantly associated with greater age, lymphovascular invasion of bladder cancer and lower 5-year overall survival. On the other hand, there are studies that have not reported a significant impact of the iPCa on the survival of patients undergoing RC, thus making this a still controversial topic [105]. Several multi-institutional series have found a biochemical recurrence rate in 3% of patients with iPCa who underwent RC, and only in rare cases was prostate cancer the cause of death in these patients [106,107,108,109]. Furthermore, in the retrospective study by Chessa et al. [110], 18% of patients died from bladder cancer, indicating that most iPCa were clinically insignificant, and the cancer outcomes of patients with iPCa undergoing CR are mainly driven by bladder cancer prognosis. These results indicate that a nerve-sparing surgical approach can be offered to patients with organ-confined bladder cancer and previous diagnosis of iPCa, ensuring a low biochemical recurrence rate without compromising patients’ life expectancy [110].

Certainly, further studies with a longer follow-up are needed to confirm these results to evaluate the real impact that a diagnosis of iPCa can have on the overall survival of the patient with BC and on the choice of the most suitable therapeutic approach.

Isolated tumor recurrence within the pouch is very rare. In these cases, it may be necessary to perform a pouchectomy with diversion of the ileal duct, or sometimes, if the patient is unfit or refuses excision of the neobladder, adjuvant radiochemotherapy may be an alternative effective treatment [111,112,113].

## 3. Functional Outcomes

### 3.1. Renal Function

As reported by Obrecht et al. [54], renal function in terms of estimated glomerular filtration rate (eGFR) at 6–12 months after surgery was not significantly different. In their study, even venous blood gas analysis at 6 months showed a median pH value of 7.38, with only two patients (16.7%) requiring bicarbonate substitution. It is also true that in other cases it is possible to see a statistically significant increase in creatinine starting at 6 months from preoperative baseline. It is always important to rule out an underlying hydronephrosis, often related to uretero-intestinal anastomotic stenosis, which should be properly investigated and treated [57,114]. In fact the most frequent cause of renal function damage is given by uretero-ileal anastomotic strictures [115]. Overall, most patients with orthotopic neobladder underwent long-term maintenance of upper tract function with an increase in serum creatinine levels that is observed in only a small percentage (3.8%) of cases after a 5-year follow-up [116,117,118,119].

### 3.2. Continence and Neobladder Training

The neobladder represents a real continent reservoir that allows adequate urination and emptying through the urethra. However, patients should be advised of the possible risk of incontinence and urinary retention [120]. Currently, the literature has reported large differences in risk regarding the potential function of the neobladder. Chang et al. reported a prevalence of day and night complete continence at one year post surgery at a low of 22% and a high of 63% [50]. According to other studies, continence is achieved in 85–100% of patients during the day and between 60–95% at night [2,121].

In recent studies, continence rates defined as 0–1 sanitary pads per day of 88–95% after one year of surgery have also been reported [122,123,124]. Nocturnal incontinence is more frequent than diurnal incontinence in both sexes, and it is believed that this is due to the absence of the guard reflex, to a longer residence time of concentrated urine then diluted by osmosis through the mucous membrane of the neobladder and to the increased nocturnal urine production [125]. Prevalence rates of nocturnal continence are usually lower (66 to 93%) [126,127,128]. It is also true that with time, continence appears to decrease due to a reduction in muscle tone and in the number of muscle cells of the external rhabdosphincter, mainly due to age changes [126,129,130,131]. However, nerve-sparing surgical approaches would appear to improve continence, and this is valid for both sexes [130,132].

Tyritzis et al. [7], at the Karolinska Institute, reported 70 patients with rates of 12-month diurnal continence of 89% in men and 67% in women undergoing RARC and totally intracorporeal orthotopic neobladder and 12-month nocturnal continence rates of 73% in men and 67% in women. Tan et al. [133] reported 95% diurnal continence and 65% continence after a two-year follow-up, while Asimakopoulos et al. [134] reported 100% diurnal continence and 72% nocturnal continence in patients undergoing RARC and totally intracorporeal orthotopic neobladder. The finding of such varied continence rates between studies can be linked to several factors, such as the application of different surgical techniques in the reconstruction of the neobladder and the lack of standardization in the definition of continence. To overcome this, the definition and measurement of continence should in fact be based on a validated instrument, such as the ICIQ UI SF (International Consultation on Incontinence Questionnaire—Urinary Incontinence Short Form) scoring system. It would be helpful for patients to record the frequency and severity of daytime and night-time incontinence in a voiding diary as part of neobladder training as well.

After surgery, it is important that the emptying of the neobladder occurs every 2–3 h to avoid overflow incontinence [58,135]. The emptying intervals can progressively lengthen as the capacity of the neobladder increases over time, until it empties every 5–6 h during the day and only once at night, thus reaching its final functional capacity, from about 150–200 mL up to 400–500 mL in a mature neobladder [131,136,137]. It is important that the patient learns to consciously relax the sphincter and pelvic floor muscles, possibly to use a Valsalva maneuver or to exert manual pressure on the abdomen and suprapubic area to achieve complete emptying [58,138]. In the event that, despite the indicated maneuvers, a high postvoiding residue remains, the patient must be instructed about the possibility of performing intermittent self-catheterization.

Steven and Poulsen in their study reported a voiding failure rate of 15.4% and 32.9% at 6 months and 3 years of follow-up, respectively. Clean Intermittent Catheterization (CIC) can even affect 20% of men and 43% of women [124] undergoing ONB. Studer et al. reported urinary retention in 22% of their patients [129], although most of the findings from European centers report a CIC rate of approximately 3% [127]. It is believed that there are several causes for this lack of emptying: excessive length of the ileum (>60 cm) with an oversized neobladder [139], a kinking of the bladder outlet [129] and stenosis of the urethra–bladder anastomosis [119,140]. The literature also shows a higher rate of urinary retention in women (21–61%), probably linked to alterations in urethral innervation or relaxation of the pelvic floor [141].

### 3.3. Potency and Sexual Function

The alteration of sexual function following radical cystectomy is an important element to be taken into consideration. Preservation of neurovascular bundles bilaterally can lead to a 60% potency rate in the two-year follow-up [142]. Asimakopoulos et al. found a return to preoperative IIEF-6 score in 72.5% of patients 12 months after surgery [57,134]. In a study conducted by Tyritzis et al., 81.2% of the nerve-spared patients were potent with or without PDE5 medication at 12 months [7]. It would seem that the choice of the type of urinary derivation is an entirely independent factor and does not affect the risk of impotence and alterations in sexual desire after surgery [143], but there are conflicting results in this regard. According to another study, the reconstruction of the neobladder would lead to a better preservation of sexual function compared with an ileal duct, with 35% of men undergoing ONB versus 9.8% by means of men undergoing the creation of an ileal duct [144]. Results were obtained using the International Erectile Function Index questionnaire.

In recent years, there has been a particular focus on the deterioration of sexual function in women after radical cystectomy [145,146]. The most frequently encountered alterations are decreased vaginal lubrication and sexual desire, anorgasmia and dyspareunia. Tuderti et al. [147] evaluated sexual function in 11 female patients with disease ≤cT2 and absence of bladder neck, trigone or urethral cancer, undergoing sex-sparing RARC with intracorporeal ONB, employing the female sexual function index questionnaire, which includes six sexual domains. Significant changes in the sexual sphere were evident three months after the operation, with a progressive improvement in the following months and with 73% of patients returning to be sexually active at an evaluation at 12 months. Thus, acceptable sexual function can be achieved in females after RARC, particularly by trying to preserve the urethra, clitoral vascularity and innervation and the anterior vaginal wall [115,132,148].

Overall, in both men and women, postoperative sexuality is correlated not only with the nerve-sparing surgical technique [149] but also with other factors such as preoperative erectile function, oncological outcome, comorbidities, urinary continence, psychological state and the skills of the surgeon [7,150].

### 3.4. Quality of Life

RC represents a surgical intervention with a high impact on the quality of life of patients due to the significant changes in urinary and sexual activity and also in general and psycho-social well-being. In this regard, several validated questionnaires have been proposed to evaluate QoL after RC and UD. A questionnaire that allows an effective assessment of the quality of life of these patients must take into account at least three aspects: the general state of health, physical and mental health and organ-specific function (urinary and sexual) after local treatment [115]. Among the most accurate validated questionnaires used to evaluate QoL after cystectomy and urinary diversion are the Short Form-12 (SF-12, the short version of the SF-36 questionnaire) [151], the European Organization for Research and Treatment QLQ-C-30 [152] (generic cancer), the EORTC QLQ-BLM-30 [153] (cancer-specific invasive bladder muscle) and the Functional Assessment of Cancer Therapy–Bladder Cancer (FACT-Bl) [154].

Several aspects affect QoL after CR: urinary incontinence, urinary retention with the need for self-catheterization, the risk of urinary infections, possibly associated intestinal disorders and the impact of the stoma, if present, on one’s body image [121,155]. The impact on QoL is also believed to depend on the type of urinary derivation and, specifically, some studies believe that ONB would provide better QoL than ileal conduit [3,88,144,155,156,157]. On the other hand, other studies that have compared ONB patients with different types of urinary diversion have not confirmed the superiority of a type of reconstruction with respect to QoL but have shown the achievement of a certain degree of well-being with any type of derivation [158,159,160,161,162,163]. In the study conducted by Autorino et al. [164] on 88 patients who used the SF 36 questionnaire, there was no statistically significant difference in the scoring parameters between the neobladder and ileal conduit groups. However, patients with neobladder who were 65 years old or older (*n* = 18) had significantly lower scores for the role in physical functioning and for emotional–social functioning than those younger than 65 years (*n* = 17; *p* < 0.05). Studies using other QoL questionnaires, such as the QoL questionnaire C30 (QLQ C30) and the muscle invasive BC module QLQ (European Organization for Research and Treatment of Cancer Instruments) (QLQ BLM), also reported similar results [88,165,166]. Therefore, at present, it remains controversial whether ONB reconstruction is superior to ICD with regards to health-related QOL (HRQOL).

Few studies have evaluated QoL in female patients undergoing CR and UD [167,168,169]. Gacci et al. demonstrated in their study that women undergoing ureterocutaneostomy have worse QoL than patients undergoing Bricker urinary shunt and ONB [170]. The most influencing element of QoL in women after CR and UD is incontinence, in particular at night, with a negative impact in all social life areas [167,168].

A significant proportion of patients after orthotopic bladder replacement suffer from sexual dysfunction [171]. Sexual activity had a notable impact on QoL after RC and ON, as did urinary incontinence, leading to a worse QoL; therefore, urologists should evaluate this disorder before and after surgery to resolve it [172]. Takenanka et al. found that although GH–QOL was generally well maintained 5 years after orthotopic neobladder replacement, the presence of CIC or daytime incontinence impaired GH–QOL [173]. In general, the impact of surgical complications and urinary diversion on QoL is greater in the postoperative period as patients are able to gradually adapt to their new condition after surgery, accepting the psychological and social implications. Certainly, age, urinary incontinence, sexual dysfunction and associated comorbidities must be carefully considered in the patient’s pre- and postoperative consultation [174].

## 4. Conclusions

After many years of clinical experience and improvements in surgical techniques, the orthotopic neobladder reconstruction following radical cystectomy has been shown to provide adequate long-term survival and low rates of local recurrence, while not compromising oncologic outcomes. The neobladder also provides excellent functional outcomes, improving patient’s quality of life. Adequate patient selection that takes into consideration tumor features, preoperative sphincter function, any comorbidities and postoperative issues that may affect patients’ quality of life is also important. A careful counseling between physician and patient should focus on several considerations regarding functional outcomes and postoperative management: metabolic complications, urinary incontinence, sexual function and an adequate patient training in neobladder management. Following these indications, the neobladder can be an excellent and safe surgical alternative in patients with bladder cancer as compared with other urinary diversion techniques.

## Figures and Tables

**Table 1:** Main Contraindications for ONB Reconstruction.

Renal and liver impairment Debilitating neurological and psychiatric pathologies Inflammatory bowel disease Positive surgical margins for urothelial carcinoma Metastatic disease Inability to adhere to neobladder training Limited life expectancy Pelvic radiotherapy Complex urethral stricture Severe urethral sphincter incontinence Lack of compliance to regular follow-up

## Abbreviations

**RC**	Radical Cystectomy
**ONB**	Orthotopic Neobladder
**UD**	Urinary Diversion
**QoL**	Quality of Life
**IC**	Ileal Conduit
**BMI**	Body Mass Index
**CT**	Computed Tomography
**FTR**	Failure To Rescue
**THCs**	Total Hospital Charges
**RARC**	Robotic-Assisted Radical Cystectomy
**VTE**	Venous Thromboembolism
**ERAS**	Enhanced Recovery After Surgery
**POI**	Postoperative Ileus
**LOS**	Length Of Stay
**ECUD**	Extracorporeal Urinary Diversion
**ORC**	Open Radical Cystectomy
**ICUD**	Intracorporeal Urinary Diversion
**RFS**	Recurrence-Free Survival
**OS**	Overall Survival
**AEs**	Adverse Events
**CSS**	Cancer-Specific Survival
**RAZOR**	Randomized Open versus Robotic Cystectomy
**PSM**	Positive Surgical Margin
**DSS**	Disease-Specific Survival
**iPCa**	Incidental Prostatic Cancer
**RCTs**	Randomized Controlled Trials
**CIC**	Clean Intermittent Catheterization
**PDE5**	Phosphodiesterase-5 Inhibitors
**GH–QOL**	General Health–Quality of Life

## References

[1] Witjes J.A., Bruins H.M., Cathomas R., Compérat E.M., Cowan N.C., Gakis G., Hernández V., Linares Espinós E., Lorch A., Neuzillet Y. (2021). European Association of Urology Guidelines on Muscle-invasive and Metastatic Bladder Cancer: Summary of the 2020 Guidelines. Eur. Urol..

[2] Lee R.K., Abol-Enein H., Artibani W., Bochner B., Dalbagni G., Daneshmand S., Fradet Y., Hautmann R.E., Lee C.T., Lerner S.P. (2014). Urinary diversion after radical cystectomy for bladder cancer: Options, patient selection, and outcomes. BJU Int..

[3] Dutta S.C., Chang S.C., Coffey C.S., Smith J.A., Jack G., Cookson M.S. (2002). Health related quality of life assessment after radical cystectomy: Comparison of ileal conduit with continent orthotopic neobladder. J. Urol..

[4] Hautmann R.E., de Petriconi R.C., Volkmer B.G. (2010). Lessons learned from 1,000 neobladders: The 90-day complication rate. J. Urol..

[5] Hautmann R.E., Volkmer B.G., Schumacher M.C., Gschwend J.E., Studer U.E. (2006). Long-term results of standard procedures in urology: The ileal neobladder. World J. Urol..

[6] Stein J.P., Ginsberg D.A., Skinner D.G. (2002). Indications and technique of the orthotopic neobladder in women. Urol. Clin. North Am..

[7] Tyritzis S.I., Hosseini A., Collins J., Nyberg T., Jonsson M.N., Laurin O., Khazaeli D., Adding C., Schumacher M., Wiklund N.P. (2013). Oncologic, Functional, and Complications Outcomes of Robot-assisted Radical Cystectomy with Totally Intracorporeal Neobladder Diversion. Eur. Urol..

[8] Qu L.G., Lawrentschuk N. (2019). Orthotopic Neobladder Reconstruction: Patient Selection And Perspectives. Res. Rep. Urol..

[9] Studer U.E., Varol C., Danuser H. (2004). Orthotopic ileal neobladder. BJU Int..

[10] Studer U.E., Hautmann R.E., Hohenfellner M., Mills R.D., Okada Y., Rowland R.G., Tobisu K., Tsukamoto T. (1998). Indications for continent diversion after cystectomy and factors affecting long-term results. Urol. Oncol. Semin. Orig. Investig..

[11] Kassouf W., Spiess P.E., Brown G.A., Liu P., Grossman H.B., Dinney C.P.N., Kamat A.M. (2008). Prostatic Urethral Biopsy Has Limited Usefulness in Counseling Patients Regarding Final Urethral Margin Status During Orthotopic Neobladder Reconstruction. J. Urol..

[12] Sathianathen N.J., Jarosek S., Lawrentschuk N., Bolton D., Konety B.R. (2019). A Simplified Frailty Index to Predict Outcomes After Radical Cystectomy. Eur. Urol. Focus.

[13] Nunzio C.D., Cicione A., Izquierdo L., Lombardo R., Tema G., Lotrecchiano G., Minervini A., Simone G., Cindolo L., D’Orta C. (2019). Multicenter Analysis of Postoperative Complications in Octogenarians After Radical Cystectomy and Ureterocutaneostomy: The Role of the Frailty Index. Clin. Genitourin. Cancer.

[14] Palumbo C., Knipper S., Pecoraro A., Rosiello G., Luzzago S., Deuker M., Tian Z., Shariat S.F., Simeone C., Briganti A. (2020). Patient frailty predicts worse perioperative outcomes and higher cost after radical cystectomy. Surg. Oncol..

[15] Clegg A., Young J., Iliffe S., Rikkert M.O., Rockwood K. (2013). Frailty in elderly people. Lancet Lond. Engl..

[16] Alvarez-Nebreda M.L., Bentov N., Urman R.D., Setia S., Huang J.C.-S., Pfeifer K., Bennett K., Ong T.D., Richman D., Gollapudi D. (2018). Recommendations for Preoperative Management of Frailty from the Society for Perioperative Assessment and Quality Improvement (SPAQI). J. Clin. Anesth..

[17] Hosseini A., Ebbing J., Collins J. (2019). Clinical outcomes of robot-assisted radical cystectomy and continent urinary diversion. Scand. J. Urol..

[18] Collins J.W., Patel H., Adding C., Annerstedt M., Dasgupta P., Khan S.M., Artibani W., Gaston R., Piechaud T., Catto J.W. (2016). Enhanced Recovery After Robot-assisted Radical Cystectomy: EAU Robotic Urology Section Scientific Working Group Consensus View. Eur. Urol..

[19] Pruthi R.S., Nielsen M., Smith A., Nix J., Schultz H., Wallen E.M. (2010). Fast Track Program in Patients Undergoing Radical Cystectomy: Results in 362 Consecutive Patients. J. Am. Coll. Surg..

[20] Wolf J.S., Bennett C.J., Dmochowski R.R., Hollenbeck B.K., Pearle M.S., Schaeffer A.J. (2008). Best Practice Policy Statement on Urologic Surgery Antimicrobial Prophylaxis. J. Urol..

[21] Chiang H.A., Cheng P.J., Speed J.M., Steinberg J., Najjar P.A., Steele G.S., Trinh Q.-D., Eswara J.R., Chang S.L., Kibel A.S. (2020). Implementation of a Perioperative Venous Thromboembolism Prophylaxis Program for Patients Undergoing Radical Cystectomy on an Enhanced Recovery After Surgery Protocol. Eur. Urol. Focus.

[22] Su S., Gu L., Ma X., Li H., Wang B., Shi T., Zhang X. (2019). Comparison of Laparoscopic and Robot-assisted Radical Cystectomy for Bladder Cancer: Perioperative and Oncologic Outcomes. Clin. Genitourin. Cancer.

[23] Xiao J., Wang M., He W., Wang J., Yang F., Ma X.-Y., Zang Y., Yang C.-G., Yu G., Wang Z.-H. (2019). Does Postoperative Rehabilitation for Radical Cystectomy Call for Enhanced Recovery after Surgery? A Systematic Review and Meta-analysis. Curr. Med. Sci..

[24] Tyson M.D., Chang S.S. (2016). Enhanced Recovery Pathways Versus Standard Care After Cystectomy: A Meta-analysis of the Effect on Perioperative Outcomes. Eur. Urol..

[25] Feng D., Liu S., Lu Y., Wei W., Han P. (2020). Clinical efficacy and safety of enhanced recovery after surgery for patients treated with radical cystectomy and ileal urinary diversion: A systematic review and meta-analysis of randomized controlled trials. Transl. Androl. Urol..

[26] Nicholson A., Lowe M.C., Parker J., Lewis S.R., Alderson P., Smith A.F. (2014). Systematic review and meta-analysis of enhanced recovery programmes in surgical patients. Br. J. Surg..

[27] Cerantola Y., Valerio M., Persson B., Jichlinski P., Ljungqvist O., Hubner M., Kassouf W., Muller S., Baldini G., Carli F. (2013). Guidelines for perioperative care after radical cystectomy for bladder cancer: Enhanced Recovery After Surgery (ERAS(^®^)) society recommendations. Clin. Nutr. Edinb. Scotl..

[28] Chang S.S., Bochner B.H., Chou R., Dreicer R., Kamat A.M., Lerner S.P., Lotan Y., Meeks J.J., Michalski J.M., Morgan T.M. (2017). Treatment of Non-Metastatic Muscle-Invasive Bladder Cancer: AUA/ASCO/ASTRO/SUO Guideline. J. Urol..

[29] Baack Kukreja J.E., Messing E.M., Shah J.B. (2016). Are we doing “better”? The discrepancy between perception and practice of enhanced recovery after cystectomy principles among urologic oncologists. Urol. Oncol..

[30] Schiavina R., Droghetti M., Bianchi L., Ercolino A., Chessa F., Casablanca C., Piazza P., Mottaran A., Recenti D., Salvador M. (2021). The robotic approach improves the outcomes of ERAS protocol after radical cystectomy: A prospective case-control analysis. Urol. Oncol..

[31] Saar M., Ohlmann C.-H., Siemer S., Lehmann J., Becker F., Stöckle M., Kamradt J. (2013). Fast-track rehabilitation after robot-assisted laparoscopic cystectomy accelerates postoperative recovery. BJU Int..

[32] Maffezzini M., Campodonico F., Capponi G., Manuputty E., Gerbi G. (2012). Fast-track surgery and technical nuances to reduce complications after radical cystectomy and intestinal urinary diversion with the modified Indiana pouch. Surg. Oncol..

[33] Baack Kukreja J.E., Kiernan M., Schempp B., Siebert A., Hontar A., Nelson B., Dolan J., Noyes K., Dozier A., Ghazi A. (2017). Quality Improvement in Cystectomy Care with Enhanced Recovery (QUICCER) study. BJU Int..

[34] Karl A., Buchner A., Becker A., Staehler M., Seitz M., Khoder W., Schneevoigt B., Weninger E., Rittler P., Grimm T. (2014). A new concept for early recovery after surgery for patients undergoing radical cystectomy for bladder cancer: Results of a prospective randomized study. J. Urol..

[35] Ziegelmueller B.K., Jokisch J.-F., Buchner A., Grimm T., Kretschmer A., Schulz G.B., Stief C., Karl A. (2020). Long-Term Follow-Up and Oncological Outcome of Patients Undergoing Radical Cystectomy for Bladder Cancer following an Enhanced Recovery after Surgery (ERAS) Protocol: Results of a Large Randomized, Prospective, Single-Center Study. Urol. Int..

[36] Moschini M., Stabile A., Mattei A., Montorsi F., Cathelineau X., Sanchez-Salas R. (2019). Enhanced recovery after surgery (ERAS) in radical cystectomy patients: From consensus to evidences. Int. Braz. J. Urol. Off. J. Braz. Soc. Urol..

[37] Guleser A.S., Basaga Y. (2022). ERAS vs. Traditional Protocol in Patients Who Had Radical Cystectomy with Ileal Conduit: A Retrospective Comparative Analysis of 182 Cases. Adv. Urol..

[38] Zhang X., Yang J., Chen X., Du L., Li K., Zhou Y. (2020). Enhanced recovery after surgery on multiple clinical outcomes: Umbrella review of systematic reviews and meta-analyses. Medicine.

[39] Xu W., Daneshmand S., Bazargani S.T., Cai J., Miranda G., Schuckman A.K., Djaladat H. (2015). Postoperative Pain Management after Radical Cystectomy: Comparing Traditional versus Enhanced Recovery Protocol Pathway. J. Urol..

[40] Lee C.T., Chang S.S., Kamat A.M., Amiel G., Beard T.L., Fergany A., Karnes R.J., Kurz A., Menon V., Sexton W.J. (2014). Alvimopan accelerates gastrointestinal recovery after radical cystectomy: A multicenter randomized placebo-controlled trial. Eur. Urol..

[41] Kouba E.J., Wallen E.M., Pruthi R.S. (2007). Gum chewing stimulates bowel motility in patients undergoing radical cystectomy with urinary diversion. Urology.

[42] Wessels F., Lenhart M., Kowalewski K.F., Braun V., Terboven T., Roghmann F., Michel M.S., Honeck P., Kriegmair M.C. (2020). Early recovery after surgery for radical cystectomy: Comprehensive assessment and meta-analysis of existing protocols. World J. Urol..

[43] Pillai P., McEleavy I., Gaughan M., Snowden C., Nesbitt I., Durkan G., Johnson M., Cosgrove J., Thorpe A. (2011). A double-blind randomized controlled clinical trial to assess the effect of Doppler optimized intraoperative fluid management on outcome following radical cystectomy. J. Urol..

[44] Svanfeldt M., Thorell A., Hausel J., Soop M., Rooyackers O., Nygren J., Ljungqvist O. (2007). Randomized clinical trial of the effect of preoperative oral carbohydrate treatment on postoperative whole-body protein and glucose kinetics. Br. J. Surg..

[45] Kehlet H. (1997). Multimodal approach to control postoperative pathophysiology and rehabilitation. Br. J. Anaesth..

[46] Krasnow R.E., Mossanen M., Koo S., Kubiak D.W., Preston M.A., Chung B.I., Kibel A.S., Chang S.L. (2017). Prophylactic Antibiotics and Postoperative Complications of Radical Cystectomy: A Population Based Analysis in the United States. J. Urol..

[47] Sung L.H., Yuk H.D. (2020). Enhanced recovery after surgery of patients undergoing radical cystectomy for bladder cancer. Transl. Androl. Urol..

[48] Frees S.K., Aning J., Black P., Struss W., Bell R., Chavez-Munoz C., Gleave M., So A.I. (2018). A prospective randomized pilot study evaluating an ERAS protocol versus a standard protocol for patients treated with radical cystectomy and urinary diversion for bladder cancer. World J. Urol..

[49] Gakis G., Stenzl A. (2010). Ileal Neobladder and Its Variants. Eur. Urol. Suppl..

[50] Chang D.T.S., Lawrentschuk N. (2015). Orthotopic neobladder reconstruction. Urol. Ann..

[51] Menon M., Hemal A.K., Tewari A., Shrivastava A., Shoma A.M., El-Tabey N.A., Shaaban A., Abol-Enein H., Ghoneim M.A. (2003). Nerve-sparing robot-assisted radical cystoprostatectomy and urinary diversion. BJU Int..

[52] Novara G., Catto J.W.F., Wilson T., Annerstedt M., Chan K., Murphy D.G., Motttrie A., Peabody J.O., Skinner E.C., Wiklund P.N. (2015). Systematic Review and Cumulative Analysis of Perioperative Outcomes and Complications After Robot-assisted Radical Cystectomy. Eur. Urol..

[53] Parekh D.J., Reis I.M., Castle E.P., Gonzalgo M.L., Woods M.E., Svatek R.S., Weizer A.Z., Konety B.R., Tollefson M., Krupski T.L. (2018). Robot-assisted radical cystectomy versus open radical cystectomy in patients with bladder cancer (RAZOR): An open-label, randomised, phase 3, non-inferiority trial. Lancet Lond. Engl..

[54] Obrecht F., Youssef N.A., Burkhardt O., Schregel C., Randazzo M., Padevit C., Wiklund P., John H. (2020). Robot-assisted radical cystectomy and intracorporeal orthotopic neobladder: 1-year functional outcomes. Asian J. Androl..

[55] Gu Q., Xia J., Xu A., Zhang T., Wang Z. (2020). Robot-assisted radical cystectomy with totally intracorporeal neobladder diversion: Perioperative, oncologic, and functional outcomes. Transl. Androl. Urol..

[56] Simone G., Papalia R., Misuraca L., Tuderti G., Minisola F., Ferriero M., Vallati G., Guaglianone S., Gallucci M. (2018). Robotic Intracorporeal Padua Ileal Bladder: Surgical Technique, Perioperative, Oncologic and Functional Outcomes. Eur. Urol..

[57] Benamran D., Phé V., Drouin S.J., Perrot O., Grégoris A., Parra J., Vaessen C., Seisen T., Rouprêt M. (2020). Functional outcomes obtained with intracorporeal neobladder after robotic radical cystectomy for cancer: A narrative review. J. Robot. Surg..

[58] Herdiman O., Ong K., Johnson L., Lawrentschuk N. (2013). Orthotopic Bladder Substitution (Neobladder): Part II: Postoperative Complications, Management, and Long-term Follow-up. J. Wound. Ostomy Cont. Nurs..

[59] Hautmann R.E., De Petriconi R.C., Volkmer B.G. (2011). 25 Years of Experience With 1000 Neobladders: Long-Term Complications. J. Urol..

[60] Bazargani S.T., Djaladat H., Ahmadi H., Miranda G., Cai J., Schuckman A.K., Daneshmand S. (2018). Gastrointestinal Complications Following Radical Cystectomy Using Enhanced Recovery Protocol. Eur. Urol. Focus.

[61] Djaladat H., Daneshmand S. (2016). Gastrointestinal Complications in Patients Who Undergo Radical Cystectomy with Enhanced Recovery Protocol. Curr. Urol. Rep..

[62] Kauf T.L., Svatek R.S., Amiel G., Beard T.L., Chang S.S., Fergany A., Karnes R.J., Koch M., O’Hara J., Lee C.T. (2014). Alvimopan, a peripherally acting μ-opioid receptor antagonist, is associated with reduced costs after radical cystectomy: Economic analysis of a phase 4 randomized, controlled trial. J. Urol..

[63] Darouiche R.O., Wall M.J., Itani K.M.F., Otterson M.F., Webb A.L., Carrick M.M., Miller H.J., Awad S.S., Crosby C.T., Mosier M.C. (2010). Chlorhexidine-Alcohol versus Povidone-Iodine for Surgical-Site Antisepsis. N. Engl. J. Med..

[64] Anderson C.B., McKiernan J.M. (2018). Surgical Complications of Urinary Diversion. Urol. Clin. N. Am..

[65] Haider M., Ladurner C., Mayr R., Tandogdu Z., Fritsche H.-M., Fradet V., Comploj E., Pycha A., Lemire F., Lacombe L. (2019). Use and duration of antibiotic prophylaxis and the rate of urinary tract infection after radical cystectomy for bladder cancer: Results of a multicentric series. Urol. Oncol..

[66] Schiavina R., Borghesi M., Guidi M., Vagnoni V., Zukerman Z., Pultrone C., Passaretti G., Romagnoli D., Bianchi L., Morselli-Labate A. (2013). Perioperative complications and mortality after radical cystectomy when using a standardized reporting methodology. Clin. Genitourin. Cancer.

[67] Takada N., Abe T., Shinohara N., Sazawa A., Maruyama S., Shinno Y., Sato S., Mitsuhashi K., Sato T., Sugishita K. (2012). Peri-operative morbidity and mortality related to radical cystectomy: A multi-institutional retrospective study in Japan. BJU Int..

[68] Mattei A., Birkhaeuser F.D., Baermann C., Warncke S.H., Studer U.E. (2008). To stent or not to stent perioperatively the ureteroileal anastomosis of ileal orthotopic bladder substitutes and ileal conduits? Results of a prospective randomized trial. J. Urol..

[69] Mullins J.K., Guzzo T.J., Ball M.W., Pierorazio P.M., Eifler J., Jarrett T.W., Schoenberg M.P., Bivalacqua T.J. (2012). Ureteral stents placed at the time of urinary diversion decreases postoperative morbidity. Urol. Int..

[70] Novara G., De Marco V., Aragona M., Boscolo-Berto R., Cavalleri S., Artibani W., Ficarra V. (2009). Complications and mortality after radical cystectomy for bladder transitional cell cancer. J. Urol..

[71] Varkarakis I.M., Chrisofos M., Antoniou N., Papatsoris A., Deliveliotis C. (2007). Evaluation of findings during re-exploration for obstructive ileus after radical cystectomy and ileal-loop urinary diversion: Insight into potential technical improvements. BJU Int..

[72] Quek M.L., Stein J.P., Daneshmand S., Miranda G., Thangathurai D., Roffey P., Skinner E.C., Lieskovsky G., Skinner D.G. (2006). A critical analysis of perioperative mortality from radical cystectomy. J. Urol..

[73] Hosseini A., Dey L., Laurin O., Adding C., Hoijer J., Ebbing J., Collins J.W. (2018). Ureteric stricture rates and management after robot-assisted radical cystectomy: A single-centre observational study. Scand. J. Urol..

[74] Moeen A.M., Safwat A.S., Elderwy A.A., Behnsawy H.M., Osman M.M., Hameed D.A. (2017). Management of neobladder complications: Endoscopy comes first. Scand. J. Urol..

[75] Razvi H.A., Martin T.V., Sosa E.R., Vaughan E.D. (1996). Endourologic management of complications of urinary intestinal diversions. AUA Update Ser..

[76] Kramolowsky E.V., Clayman R.V., Weyman P.J. (1987). Endourological management of ureteroileal anastomotic strictures: Is it effective?. J. Urol..

[77] Słojewski M., Sikorski A., Gołab A. (2000). Giant neobladder calculus. Scand. J. Urol. Nephrol..

[78] Blyth B., Ewalt D.H., Duckett J.W., Snyder H.M. (1992). Lithogenic properties of enterocystoplasty. J. Urol..

[79] Boyd S.D., Everett R.W., Schiff W.M., Fugelso P.D. (1988). Treatment of unusual Kock pouch urinary calculi with extracorporeal shock wave lithotripsy. J. Urol..

[80] Madbouly K. (2010). Large orthotopic reservoir stone burden: Role of open surgery. Urol. Ann..

[81] L’Esperance J.O., Sung J., Marguet C., L’Esperance A., Albala D.M. (2004). The surgical management of stones in patients with urinary diversions. Curr. Opin. Urol..

[82] Ma J.L., Hennessey D.B., Newell B.P., Bolton D.M., Lawrentschuk N. (2018). Radiotherapy-related complications presenting to a urology department: A more common problem than previously thought?. BJU Int..

[83] Kim K.H., Yoon H.S., Yoon H., Chung W.S., Sim B.S., Ryu D.-R., Lee D.H. (2016). Risk Factors for Developing Metabolic Acidosis after Radical Cystectomy and Ileal Neobladder. PLoS ONE.

[84] Wiederkehr M., Krapf R. (2001). Metabolic and endocrine effects of metabolic acidosis in humans. Swiss Med. Wkly..

[85] Stein R., Schröder A., Thüroff J.W. (2012). Bladder augmentation and urinary diversion in patients with neurogenic bladder: Non-surgical considerations. J. Pediatr. Urol..

[86] Turk T.M.T., Koleski F.C., Albala D.M. (1999). Incidence of urolithiasis in cystectomy patients after intestinal conduit or continent urinary diversion. World J. Urol..

[87] Wullt B., Agace W., Mansson W. (2004). Bladder, bowel and bugs—bacteriuria in patients with intestinal urinary diversion. World J. Urol..

[88] Erber B., Schrader M., Miller K., Schostak M., Baumunk D., Lingnau A., Schrader A.J., Jentzmik F. (2012). Morbidity and Quality of Life in Bladder Cancer Patients following Cystectomy and Urinary Diversion: A Single-Institution Comparison of Ileal Conduit versus Orthotopic Neobladder. ISRN Urol..

[89] Thorstenson A., Jacobsson H., Onelöv E., Holst J.J., Hellström P.M., Kinn A.-C. (2007). Gastrointestinal function and metabolic control after construction of an orthotopic ileal neobladder in bladder cancer. Scand. J. Urol. Nephrol..

[90] Sonpavde G., Khan M.M., Lerner S.P., Svatek R.S., Novara G., Karakiewicz P.I., Skinner E., Tilki D., Kassouf W., Fradet Y. (2011). Disease-Free Survival at 2 or 3 Years Correlates With 5-Year Overall Survival of Patients Undergoing Radical Cystectomy for Muscle Invasive Bladder Cancer. J. Urol..

[91] Raza S.J., Wilson T., Peabody J.O., Wiklund P., Scherr D.S., Al-Daghmin A., Dibaj S., Khan M.S., Dasgupta P., Mottrie A. (2015). Long-term Oncologic Outcomes Following Robot-assisted Radical Cystectomy: Results from the International Robotic Cystectomy Consortium. Eur. Urol..

[92] Venkatramani V., Reis I.M., Castle E.P., Gonzalgo M.L., Woods M.E., Svatek R.S., Weizer A.Z., Konety B.R., Tollefson M., Krupski T.L. (2020). Predictors of Recurrence, and Progression-Free and Overall Survival following Open versus Robotic Radical Cystectomy: Analysis from the RAZOR Trial with a 3-Year Followup. J. Urol..

[93] Wilson T.G., Guru K., Rosen R.C., Wiklund P., Annerstedt M., Bochner B.H., Chan K.G., Montorsi F., Mottrie A., Murphy D. (2015). Best Practices in Robot-assisted Radical Cystectomy and Urinary Reconstruction: Recommendations of the Pasadena Consensus Panel. Eur. Urol..

[94] Collins J.W., Hosseini A., Adding C., Nyberg T., Koupparis A., Rowe E., Perry M., Issa R., Schumacher M.C., Wijburg C. (2017). Early Recurrence Patterns Following Totally Intracorporeal Robot-assisted Radical Cystectomy: Results from the EAU Robotic Urology Section (ERUS) Scientific Working Group. Eur. Urol..

[95] Dotan Z.A., Kavanagh K., Yossepowitch O., Kaag M., Olgac S., Donat M., Herr H.W. (2007). Positive surgical margins in soft tissue following radical cystectomy for bladder cancer and cancer specific survival. J. Urol..

[96] Novara G., Svatek R.S., Karakiewicz P.I., Skinner E., Ficarra V., Fradet Y., Lotan Y., Isbarn H., Capitanio U., Bastian P.J. (2010). Soft tissue surgical margin status is a powerful predictor of outcomes after radical cystectomy: A multicenter study of more than 4,400 patients. J. Urol..

[97] Picozzi S., Ricci C., Gaeta M., Ratti D., Macchi A., Casellato S., Bozzini G., Carmignani L. (2012). Upper urinary tract recurrence following radical cystectomy for bladder cancer: A meta-analysis on 13,185 patients. J. Urol..

[98] Claps F., van de Kamp M.W., Mayr R., Bostrom P.J., Boormans J.L., Eckstein M., Mertens L.S., Boevé E.R., Neuzillet Y., Burger M. (2021). Risk factors associated with positive surgical margins’ location at radical cystectomy and their impact on bladder cancer survival. World J. Urol..

[99] Kates M., Ball M.W., Chappidi M.R., Baras A.S., Gordetsky J., Sopko N.A., Brant A., Pierorazio P.M., Epstein J.I., Schoenberg M.P. (2016). Accuracy of urethral frozen section during radical cystectomy for bladder cancer. Urol. Oncol..

[100] Laukhtina E., Boehm A., Peyronnet B., Bravi C.A., Batista Da Costa J., Soria F., D’Andrea D., Rajwa P., Quhal F., Yanagisawa T. (2022). Urethrectomy at the time of radical cystectomy for non-metastatic urothelial carcinoma of the bladder: A collaborative multicenter study. World J. Urol..

[101] Hakozaki K., Kikuchi E., Ogihara K., Shigeta K., Abe T., Miyazaki Y., Kaneko G., Maeda T., Yoshimine S., Kanai K. (2021). Significance of prophylactic urethrectomy at the time of radical cystectomy for bladder cancer. Jpn. J. Clin. Oncol..

[102] Lee S.-H., Chang P.-L., Chen S.-M., Sun G.-H., Chen C.-L., Shen B.-Y., Wu Y.-S., Tsui K.-H. (2006). Synchronous primary carcinomas of the bladder and prostate. Asian J. Androl..

[103] Babaian R.J., Troncoso P., Ayala A. (1991). Transurethral-resection zone prostate cancer detected at cystoprostatectomy. A detailed histologic analysis and clinical implications. Cancer.

[104] Fahmy O., Khairul-Asri M.G., Schubert T., Renninger M., Stenzl A., Gakis G. (2017). Clinicopathological Features and Prognostic Value of Incidental Prostatic Adenocarcinoma in Radical Cystoprostatectomy Specimens: A Systematic Review and Meta-Analysis of 13,140 Patients. J. Urol..

[105] Claps F., Pavan N., Umari P., Rizzo M., Barbone F., Giangreco M., Liguori G., Mir M., Bussani R., Trombetta C. (2020). Incidence, predictive factors and survival outcomes of incidental prostate cancer in patients who underwent radical cystectomy for bladder cancer. Minerva Urol. Nefrol..

[106] Bruins H.M., Djaladat H., Ahmadi H., Sherrod A., Cai J., Miranda G., Skinner E.C., Daneshmand S. (2013). Incidental prostate cancer in patients with bladder urothelial carcinoma: Comprehensive analysis of 1476 radical cystoprostatectomy specimens. J. Urol..

[107] Malte R., Kluth L.A., Kaushik D., Boorjian S.A., Abufaraj M., Foerster B., Rink M., Gust K., Roghmann F., Noldus J. (2017). Frequency and prognostic significance of incidental prostate cancer at radical cystectomy: Results from an international retrospective study. Eur. J. Surg. Oncol. J. Eur. Soc. Surg. Oncol. Br. Assoc. Surg. Oncol..

[108] Tanaka T., Koie T., Ohyama C., Hashimoto Y., Imai A., Tobisawa Y., Hatakeyama S., Yamamoto H., Yoneyama T., Horiguchi H. (2017). Incidental prostate cancer in patients with muscle-invasive bladder cancer who underwent radical cystoprostatectomy. Jpn. J. Clin. Oncol..

[109] Pan J., Xue W., Sha J., Yang H., Xu F., Xuan H., Li D., Huang Y. (2014). Incidental prostate cancer at the time of cystectomy: The incidence and clinicopathological features in Chinese patients. PLoS ONE.

[110] Chessa F., Möller A., Collins J., Laurin O., Aly M., Schiavina R., Adding C., Distefano C., Akre O., Bertaccini A. (2021). Oncologic outcomes of patients with incidental prostate cancer who underwent RARC: A comparison between nerve sparing and non-nerve sparing approach. J. Robot. Surg..

[111] Hautmann R.E., Simon J. (1999). Ileal neobladder and local recurrence of bladder cancer: Patterns of failure and impact on function in men. J. Urol..

[112] Huguet J., Palou J., Serrallach M., Solé Balcells F.J., Salvador J., Villavicencio H. (2003). Management of urethral recurrence in patients with Studer ileal neobladder. Eur. Urol..

[113] Moeen A.M. (2015). Recurrence of isolated transitional cell carcinoma in an orthotopic ileal neobladder: A case report. Afr. J. Urol..

[114] Lantz A.G., Saltel M.E., Cagiannos I. (2010). Renal and functional outcomes following cystectomy and neobladder reconstruction. Can. Urol. Assoc. J..

[115] Minervini A., Serni S., Vittori G., Masieri L., Siena G., Lanciotti M., Lapini A., Gacci M., Carini M. (2014). Current indications and results of orthotopic ileal neobladder for bladder cancer. Expert Rev. Anticancer Ther..

[116] Minervini A., Boni G., Salinitri G., Mariani G., Minervini R. (2005). Evaluation of renal function and upper urinary tract morphology in the ileal orthotopic neobladder with no antireflux mechanism. J. Urol..

[117] Thoeny H.C., Sonnenschein M.J., Madersbacher S., Vock P., Studer U.E. (2002). Is ileal orthotopic bladder substitution with an afferent tubular segment detrimental to the upper urinary tract in the long term?. J. Urol..

[118] Hollowell C.M., Christiano A.P., Steinberg G.D. (2000). Technique of Hautmann ileal neobladder with chimney modification: Interim results in 50 patients. J. Urol..

[119] Minervini R., Pagni R., Mariani C., Morelli A., Morelli G., Minervini A. (2010). Effects on renal function of obstructive and nonobstructive dilatation of the upper urinary tract in ileal neobladders with refluxing ureteroenteric anastomoses. Eur. J. Surg. Oncol..

[120] Goldberg H., Baniel J., Mano R., Rotlevy G., Kedar D., Yossepowitch O. (2016). Orthotopic neobladder vs. ileal conduit urinary diversion: A long-term quality-of-life comparison. Urol. Oncol. Semin. Orig. Investig..

[121] Hautmann R.E., Abol-Enein H., Davidsson T., Gudjonsson S., Hautmann S.H., Holm H.V., Lee C.T., Liedberg F., Madersbacher S., Manoharan M. (2013). ICUD-EAU International Consultation on Bladder Cancer 2012: Urinary Diversion. Eur. Urol..

[122] Stenzl A., Jarolim L., Coloby P., Golia S., Bartsch G., Babjuk M., Kakizoe T., Robertson C. (2001). Urethra-sparing cystectomy and orthotopic urinary diversion in women with malignant pelvic tumors. Cancer.

[123] Gburek B.M., Lieber M.M., Blute M.L. (1998). Comparison of studer ileal neobladder and ileal conduit urinary diversion with respect to perioperative outcome and late complications. J. Urol..

[124] Steven K., Poulsen A.L. (2000). The orthotopic Kock ileal neobladder: Functional results, urodynamic features, complications and survival in 166 men. J. Urol..

[125] El Bahnasawy M.S., Osman Y., Gomha M.A., Shaaban A.A., Ashamallah A., Ghoneim M.A. (2000). Nocturnal enuresis in men with an orthotopic ileal reservoir: Urodynamic evaluation. J. Urol..

[126] Madersbacher S., Hochreiter W., Burkhard F., Thalmann G.N., Danuser H., Markwalder R., Studer U.E. (2003). Radical Cystectomy for Bladder Cancer Today—A Homogeneous Series Without Neoadjuvant Therapy. J. Clin. Oncol..

[127] Hautmann R.E., de Petriconi R., Gottfried H.W., Kleinschmidt K., Mattes R., Paiss T. (1999). The ileal neobladder: Complications and functional results in 363 patients after 11 years of followup. J. Urol..

[128] Ghoneim M.A., Shaaban A.A., Mahran M.R., Kock N.G. (1992). Further Experience with the Urethral Kock Pouch. J. Urol..

[129] Studer U.E., Burkhard F.C., Schumacher M., Kessler T.M., Thoeny H., Fleischmann A., Thalmann G.N. (2006). Twenty Years Experience With an Ileal Orthotopic Low Pressure Bladder Substitute—Lessons to be Learned. J. Urol..

[130] Strasser H., Marksteiner R., Margreiter E., Pinggera G.M., Mitterberger M., Frauscher F., Ulmer H., Fussenegger M., Kofler K., Bartsch G. (2007). RETRACTED: Autologous myoblasts and fibroblasts versus collagen for treatment of stress urinary incontinence in women: A randomised controlled trial. Lancet.

[131] Strasser H., Tiefenthaler M., Steinlechner M., Bartsch G., Konwalinka G. (1999). Urinary incontinence in the elderly and age-dependent apoptosis of rhabdosphincter cells. Lancet.

[132] Stenzl A., Colleselli K., Poisel S., Feichtinger H., Pontasch H., Bartsch G. (1995). Rationale and technique of nerve sparing radical cystectomy before an orthotopic neobladder procedure in women. J. Urol..

[133] Tan W.S., Sridhar A., Goldstraw M., Zacharakis E., Nathan S., Hines J., Cathcart P., Briggs T., Kelly J.D. (2015). Robot-assisted intracorporeal pyramid neobladder. BJU Int..

[134] Asimakopoulos A.D., Campagna A., Gakis G., Corona M.V.E., Piechaud T., Hoepffner J.-L., Mugnier C., Gaston R. (2016). Nerve Sparing, Robot-Assisted Radical Cystectomy with Intracorporeal Bladder Substitution in the Male. J. Urol..

[135] Steers W.D. (2000). Voiding dysfunction in the orthotopic neobladder. World J. Urol..

[136] Madersbacher S., Möhrle K., Burkhard F., Studer U.E. (2002). Long-term voiding pattern of patients with ileal orthotopic bladder substitutes. J. Urol..

[137] Burkhard F.C., Kessler T.M., Springer J., Studer U.E. (2006). Early and late urodynamic assessment of ileal orthotopic bladder substitutes combined with an afferent tubular segment. J. Urol..

[138] Ong K., Herdiman O., Johnson L., Lawrentschuk N. (2013). Orthotopic Bladder Substitution (Neobladder): Part I: Indications, Patient Selection, Preoperative Education, and Counseling. J. Wound. Ostomy Cont. Nurs..

[139] Studer U.E., Zingg E.J. (1997). ILEAL ORTHOTOPIC BLADDER SUBSTITUTES: What We Have Learned From 12 Years’ Experience With 200 Patients. Urol. Clin. N. Am..

[140] Pantuck A.J., Han K.-R., Perrotti M., Weiss R.E., Cummings K.B. (2000). Ureteroenteric anastomosis in continent urinary diversion: Long-term results and complications of direct versus nonrefluxing techniques. J. Urol..

[141] Kübler H., Gschwend J.E. (2011). Ileal neobladder in women with bladder cancer: Cancer control and functional aspects. Curr. Opin. Urol..

[142] Kessler T.M., Burkhard F.C., Perimenis P., Danuser H., Thalmann G.N., Hochreiter W.W., Studer U.E. (2004). Attempted nerve sparing surgery and age have a significant effect on urinary continence and erectile function after radical cystoprostatectomy and ileal orthotopic bladder substitution. J. Urol..

[143] Asgari M.A., Safarinejad M.R., Shakhssalim N., Soleimani M., Shahabi A., Amini E. (2013). Sexual Function after Non-Nerve-Sparing Radical Cystoprostatectomy: A Comparison between Ileal Conduit Urinary Diversion and Orthotopic Ileal Neobladder Substitution. Int. Braz. J. Urol..

[144] Asgari M.A., Safarinejad M.R., Shakhssalim N., Soleimani M., Shahabi A., Amini E. (2013). Quality of life after radical cystectomy for bladder cancer in men with an ileal conduit or continent urinary diversion: A comparative study. Urol. Ann..

[145] Volkmer B.G., Gschwend J.E., Herkommer K., Simon J., Küfer R., Hautmann R.E. (2004). Cystectomy and orthotopic ileal neobladder: The impact on female sexuality. J. Urol..

[146] Zippe C.D., Raina R., Shah A.D., Massanyi E.Z., Agarwal A., Ulchaker J., Jones S., Klein E. (2004). Female sexual dysfunction after radical cystectomy: A new outcome measure. Urology.

[147] Tuderti G., Mastroianni R., Flammia S., Ferriero M., Leonardo C., Anceschi U., Brassetti A., Guaglianone S., Gallucci M., Simone G. (2020). Sex-Sparing Robot-Assisted Radical Cystectomy with Intracorporeal Padua Ileal Neobladder in Female: Surgical Technique, Perioperative, Oncologic and Functional Outcomes. J. Clin. Med..

[148] Bjerre B.D., Johansen C., Steven K. (1997). A questionnaire study of sexological problems following urinary diversion in the female patient. Scand. J. Urol. Nephrol..

[149] Zippe C., Nandipati K., Agarwal A., Raina R. (2006). Sexual dysfunction after pelvic surgery. Int. J. Impot. Res..

[150] Salonia A., Castagna G., Capogrosso P., Castiglione F., Briganti A., Montorsi F. (2015). Prevention and management of post prostatectomy erectile dysfunction. Transl. Androl. Urol..

[151] Ware J.E., Kosinski M., Keller S.D. (1996). A 12-Item Short-Form Health Survey: Construction of Scales and Preliminary Tests of Reliability and Validity. Med. Care.

[152] Charlson M.E., Pompei P., Ales K.L., MacKenzie C.R. (1987). A new method of classifying prognostic comorbidity in longitudinal studies: Development and validation. J. Chronic Dis..

[153] Hjermstad M.J., Fossa S.D., Bjordal K., Kaasa S. (1995). Test/retest study of the European Organization for Research and Treatment of Cancer Core Quality-of-Life Questionnaire. J. Clin. Oncol..

[154] Månsson Å., Davidsson T., Hunt S., Månsson W. (2002). The quality of life in men after radical cystectomy with a continent cutaneous diversion or orthotopic bladder substitution: Is there a difference?. BJU Int..

[155] Henningsohn L., Steven K., Kallestrup E.B., Steineck G. (2002). Distressful Symptoms and Well-being After Radical Cystectomy and Orthotopic Bladder Substitution Compared With a Matched Control Population. J. Urol..

[156] Hobisch A., Tosun K., Kinzl J., Kemmler G., Bartsch G., Höltl L., Stenzl A. (2001). Life after cystectomy and orthotopic neobladder versus ileal conduit urinary diversion. Semin. Urol. Oncol..

[157] Shi H., Yu H., Bellmunt J., Leow J.J., Chen X., Guo C., Yang H., Zhang X. (2018). Comparison of health-related quality of life (HRQoL) between ileal conduit diversion and orthotopic neobladder based on validated questionnaires: A systematic review and meta-analysis. Qual. Life Res..

[158] Gerharz E.W. (2007). Is there any evidence that one continent diversion is any better than any other or than ileal conduit?. Curr. Opin. Urol..

[159] Gerharz E.W., Månsson A., Hunt S., Skinner E.C., Månsson W. (2005). Quality of life after cystectomy and urinary diversion: An evidence based analysis. J. Urol..

[160] Wright J.L., Porter M.P. (2007). Quality-of-life assessment in patients with bladder cancer. Nat. Clin. Pract. Urol..

[161] Porter M.P., Wei J.T., Penson D.F. (2005). Quality of life issues in bladder cancer patients following cystectomy and urinary diversion. Urol. Clin. North Am..

[162] Porter M.P., Penson D.F. (2005). Health related quality of life after radical cystectomy and urinary diversion for bladder cancer: A systematic review and critical analysis of the literature. J. Urol..

[163] Fujisawa M., Isotani S., Gotoh A., Okada H., Arakawa S., Kamidono S. (2000). Health-Related quality of life with orthotopic neobladder versus ileal conduit according to the SF-36 survey. Urology.

[164] Autorino R., Quarto G., Di Lorenzo G., De Sio M., Perdonà S., Giannarini G., Giugliano F., Damiano R. (2009). Health related quality of life after radical cystectomy: Comparison of ileal conduit to continent orthotopic neobladder. Eur. J. Surg. Oncol. J. Eur. Soc. Surg. Oncol. Br. Assoc. Surg. Oncol..

[165] Sogni F., Brausi M., Frea B., Martinengo C., Faggiano F., Tizzani A., Gontero P. (2008). Morbidity and quality of life in elderly patients receiving ileal conduit or orthotopic neobladder after radical cystectomy for invasive bladder cancer. Urology.

[166] Singh V., Yadav R., Sinha R.J., Gupta D.K. (2014). Prospective comparison of quality-of-life outcomes between ileal conduit urinary diversion and orthotopic neobladder reconstruction after radical cystectomy: A statistical model. BJU Int..

[167] Zahran M.H., El-Hefnawy A.S., Zidan E.M., El-Bilsha M.A., Taha D.-E., Ali-El-Dein B. (2014). Health-related quality of life after radical cystectomy and neobladder reconstruction in women: Impact of voiding and continence status. Int. J. Urol. Off. J. Jpn. Urol. Assoc..

[168] Rouanne M., Legrand G., Neuzillet Y., Ghoneim T., Cour F., Letang N., Yonneau L., Hervé J.-M., Botto H., Lebret T. (2014). Long-term women-reported quality of life after radical cystectomy and orthotopic ileal neobladder reconstruction. Ann. Surg. Oncol..

[169] Xing W., Zeng S., Xu Z., Xing S., Liu Q. (2022). Comparison of Health-Related Quality of Life Between Ileal Conduit Diversion and Orthotopic Neobladder in Women: A Meta-Analysis. Front. Oncol..

[170] Gacci M., Saleh O., Cai T., Gore J.L., D’Elia C., Minervini A., Masieri L., Giannessi C., Lanciotti M., Varca V. (2013). Quality of life in women undergoing urinary diversion for bladder cancer: Results of a multicenter study among long-term disease-free survivors. Health Qual. Life Outcomes.

[171] Kretschmer A., Grimm T., Buchner A., Stief C.G., Karl A. (2016). Prognostic features for quality of life after radical cystectomy and orthotopic neobladder. Int. Braz. J. Urol. Off. J. Braz. Soc. Urol..

[172] Tostivint V., Verhoest G., Cabarrou B., Gas J., Coloby P., Zgheib J., Thoulouzan M., Soulié M., Gamé X., Beauval J.B. (2021). Quality of life and functional outcomes after radical cystectomy with ileal orthotopic neobladder replacement for bladder cancer: A multicentre observational study. World J. Urol..

[173] Takenaka A., Hara I., Soga H., Sakai I., Terakawa T., Muramaki M., Miyake H., Tanaka K., Fujisawa M. (2011). Assessment of long-term quality of life in patients with orthotopic neobladder followed for more than 5 years. Int. Urol. Nephrol..

[174] Imbimbo C., Mirone V., Siracusano S., Niero M., Cerruto M.A., Lonardi C., Artibani W., Bassi P., Iafrate M., Racioppi M. (2015). Quality of Life Assessment With Orthotopic Ileal Neobladder Reconstruction After Radical Cystectomy: Results From a Prospective Italian Multicenter Observational Study. Urology.

